# Anoscrotal distance and urogenital anomalies in ART-conceived male infants: a retrospective cohort study

**DOI:** 10.3389/frph.2025.1721022

**Published:** 2025-12-15

**Authors:** Elif Ganime Aygün, Edis Kahraman

**Affiliations:** Department of Obstetrics and Gynecology, Acıbadem Mehmet Ali Aydınlar University Atakent Hospital, Istanbul, Türkiye

**Keywords:** anoscrotal distance, frozen–thawed embryo transfer, hormone replacement therapy, hypospadias, natural cycle, urogenital anomalies

## Abstract

**Background:**

Assisted reproductive technology (ART) is widely used, yet potential effects on androgen-sensitive male genital development remain a concern. Anoscrotal distance (ASD), a validated marker of prenatal androgen exposure, may differ in ART-conceived infants. This study compared ASD and urogenital anomalies among male newborns conceived via frozen-thawed embryo transfer (FET) using natural-cycle (tNC) or hormone-replacement therapy (HRT) protocols versus natural conception (NC).

**Methods:**

In this retrospective cohort of 432 singleton male births (156 NC, 132 FET–tNC, 144 FET–HRT) delivered between 2021 and 2023, neonatal outcomes, including ASD, hypospadias, and undescended testes (UDT), were assessed. Group comparisons were performed using standard statistical tests, and exploratory modelling was conducted to identify variables most strongly distinguishing conception groups.

**Results:**

Median ASD was significantly longer in NC infants (26.2 mm) than in FET–tNC (24.9 mm) and FET–HRT (24.6 mm) infants (*p* < 0.001), with no difference between FET protocols. Hypospadias was less frequent in FET–HRT than FET–tNC infants (*p* = 0.031), while UDT was more common in NC than FET–tNC infants (*p* = 0.041). Exploratory modelling identified ASD as the strongest discriminator across conception groups.

**Conclusions:**

Male infants conceived via FET consistently exhibited shorter neonatal ASD than naturally conceived infants, suggesting subtle alterations in androgen-sensitive genital development. These findings underscore the importance of incorporating careful genital assessment and ART-specific counseling into neonatal care.

## 1 Introduction

Infertility represents a growing global health challenge, and assisted reproductive technology (ART)—including *in vitro* fertilization (IVF), intracytoplasmic sperm injection (ICSI), and cryopreservation—offers effective solutions when natural conception is not possible (1). While ART has revolutionized reproductive medicine, concerns remain regarding its potential influence on perinatal outcomes. Evidence suggests that ART-conceived infants may carry a modestly increased risk of congenital anomalies, with particular concern for urogenital malformations such as hypospadias and cryptorchidism (2).

Male genital development is critically dependent on adequate androgen exposure during a critical window in early gestation (3). Anoscrotal distance (ASD), defined as the distance from the center of the anus to the posterior base of the scrotum, is a validated anthropometric marker of prenatal androgen activity (4, 5). Shorter ASD has been associated with an increased risk of hypospadias and cryptorchidism in newborns, while in adults, reduced anogenital distance correlates with impaired spermatogenesis and lower circulating testosterone (6). Despite this biological relevance, relatively few studies have compared ASD between ART-conceived and naturally conceived infants, and reported findings remain inconsistent (7).

Biological and mechanistic evidence suggests plausible pathways through which ART procedures may influence fetal androgen signaling. Ovarian stimulation has been shown to alter endometrial cytokine, chemokine, and hormonal profiles, potentially modifying the early intrauterine endocrine environment (8, 9). Experimental and animal studies further demonstrate that exogenous estrogen and progesterone—similar to regimens used in hormone-replacement therapy (HRT) frozen embryo transfer (FET) cycles—can influence fetal Leydig cell activity, androgen receptor expression, and genital tubercle development, supporting a mechanistic link between ART-related hormonal environments and androgen-sensitive outcomes.

The increasing use of FET worldwide highlights the importance of addressing existing gaps in understanding its potential effects on neonatal development. FET offers several clinical advantages, including reduced ovarian hyperstimulation risk and enhanced scheduling flexibility (9–12). Nonetheless, preparation strategies differ: tNC relies on spontaneous ovulation, thereby maintaining physiological hormonal profiles, whereas HRT cycles involve exogenous estrogen and progesterone to induce endometrial receptivity. These differing hormonal exposures may plausibly influence androgenization during early fetal development, yet their implications for male genital outcomes are not fully understood.

Previous clinical studies examining ASD in ART populations are limited by several methodological issues. Variability in sample size, heterogeneity in ART protocols, inconsistent measurement techniques, and incomplete adjustment for confounders such as gestational age, birth anthropometrics, parental subfertility, and maternal metabolic or endocrine factors, likely contribute to differing results (3, 7, 10). Additional biases may arise from differential documentation of congenital anomalies or variations in obstetric management between ART and naturally conceived pregnancies. These limitations highlight the need for rigorously designed studies using standardized ASD assessment and clearly defined conception groups.

Against this background, the present study aimed to investigate ASD and the prevalence of urogenital anomalies—specifically hypospadias and undescended testes—among male infants conceived via FET–tNC, FET–HRT, and natural conception.We hypothesized that FET, regardless of preparation protocol, would be associated with shorter neonatal ASD compared with natural conception, while differences in urogenital anomaly prevalence between protocols would be minimal.

## 2 Materials and methods

This retrospective cohort study was conducted at the Assisted Reproductive Techniques Centre of Acıbadem Atakent Hospital, Türkiye, and included deliveries between January 2021 and December 2023. Ethical approval was obtained from the Acıbadem University Ethics Committee (Approval No. 2024-1/14). Owing to the retrospective nature of the study and the anonymization of all data, the requirement for informed consent was waived.

The study population consisted exclusively of singleton male newborns delivered at ≥36 weeks of gestation following one of three conception methods: (i) spontaneous natural conception (Naturally Conceived group), (ii) frozen–thawed embryo transfer performed in a true natural cycle with luteal support using oral dydrogesterone (10 mg three times daily) (FET–tNC group), or (iii) frozen–thawed embryo transfer following endometrial preparation with exogenous estrogen and subsequent combined estrogen plus dydrogesterone (FET–HRT group). Exclusion criteria applied only within male singleton births and included severe maternal chronic disease, prior chemo-radiotherapy, substance abuse, severe ovarian failure, polycystic ovary syndrome, endometriosis, and incomplete medical records.

Maternal variables collected included age at delivery and body mass index (BMI). For IVF pregnancies, transfer-day estradiol (E2), progesterone (P4), and thyroid-stimulating hormone (TSH) levels were extracted from electronic medical records. Obstetric variables included mode of delivery and gestational age, whereas neonatal variables included birth weight, birth length, and anoscrotal distance (ASD). ASD was defined as the distance from the center of the anus to the posterior base of the scrotum and was measured within 48 h after delivery using a precision neonatal sliding caliper. Infants were positioned supine with gentle hip abduction. Measurements were performed twice by two experienced clinicians, and the mean value was used for analysis. Although clinicians were not formally blinded to conception mode, a standardized measurement protocol was followed to minimize observer bias. No formal inter-observer reliability testing was conducted, which is acknowledged as a study limitation.

Secondary maternal and neonatal data (hormone levels, birth characteristics, and congenital anomaly diagnoses) were obtained from the hospital's electronic medical record (EMR) system, which undergoes routine internal data-validation audits. These data are entered contemporaneously by attending obstetricians and neonatologists, supporting their reliability despite the retrospective design.

Several clinically relevant confounders, including maternal smoking, parity, environmental exposures, paternal reproductive characteristics, and infertility etiology, were not systematically recorded in the EMR and therefore could not be incorporated into adjusted multivariable analyses.

Statistical analyses were performed using SPSS version 25.0 (IBM Corp., Armonk, NY, USA). Normality of continuous variables was examined with the Kolmogorov–Smirnov test, and homogeneity of variance with Levene's test. Given their non-normal distribution, continuous variables were compared among groups using the Kruskal–Wallis test with Dunn's *post hoc* procedure and Monte Carlo simulation. Categorical variables were compared using Pearson's *χ*^2^ test with Benjamini–Hochberg correction for multiple testing. To quantify the magnitude of between-group differences, effect-size statistics were calculated. For continuous variables, the Kruskal–Wallis–based *η*^2^ (eta-squared). *η*^2^ expresses the proportion of variance in the outcome attributable to group membership and is appropriate for non-normally distributed, three-group comparisons. For categorical variables, effect sizes were expressed as Cramer's *V*.

To evaluate whether fetal growth modified the ASD differences between conception groups, ASD was also stratified by standardized birth-weight categories (<2,500 g, 2,500–3,000 g, 3,000–3,500 g, 3,500–4,000 g, ≥4,000 g). These stratified analyses were conducted because prior evidence suggests that neonatal anthropometric indices, including ASD, may vary with growth parameters at birth (13).

For exploratory analyses, several supervised machine-learning classifiers (multilayer perceptron, random forest, decision tree, and multinomial logistic regression) were evaluated to identify variables most influential in distinguishing the three conception groups. The dataset was randomly split into a 70% training set and a 30% testing set. The multilayer perceptron model achieved the highest predictive accuracy; variable-importance scores from this model were reported. A two-sided *p*-value <0.05 was considered statistically significant.

## 3 Results

A total of 432 singleton male newborns were included in the analysis: 156 naturally conceived (NC), 132 conceived via FET–tNC, and 144 via FET–HRT. Maternal age was comparable across groups (overall mean 33.3 ± 5.2 years), while maternal BMI was significantly lower in the FET–tNC group compared with NC (*p* = 0.001; *η*^2^ = 0.032). Among IVF pregnancies, transfer-day hormonal profiles differed substantially: estradiol levels were highest in the FET–HRT group (296 pg/mL) compared with FET–tNC (181 pg/mL), and progesterone was similarly elevated in FET–HRT cycles (13.4 ng/mL vs. 10.8 ng/mL), with small–moderate effect sizes (*η*^2^ = 0.023–0.028) (Table 1).

**Table 1:** Demographic and clinical characteristics of the study population and overall neonatal outcomes.

Variable	*n*	%	Mean ± SD	Median (Min–Max)
Agent				
Naturally Conceived group (NC)	156	36.1		
FET–tNC group, Dydrogesterone	132	30.6		
FET–HRT group, Est + Dydrogesterone	144	33.3		
Mode of delivery				
Caesarean	320	74.1		
Vaginal birth	112	25.9		
Additional anomaly				
Hypospadias	91	21.1		
Undescended testis	47	10.9		
Absent	294	68.1		
Age (year)			33.3 ± 5.2	33 (20–45)
BMI (kg/m^2^)			25.9 ± 4.5	25 (17.3–41.19)
Transfer P4 (ng/mL)			20.4 ± 18.5	12.65 (2.42–79.28)
Transfer day E2 (pg/mL)			261.9 ± 100.3	271.5 (31–475)
TSH (mIU/L)			3.1 ± 1.5	2.875 (0.19–8.28)
Birth length (cm)			50.9 ± 1.5	51 (47–55)
Birth weight (g)			3,287.3 ± 263.1	3,275 (2,665–3,910)
Birth week (week)			39.6 ± 1.4	40 (36–45)
Distance (mm)			25.3 ± 1.3	25.29 (22.7–29.51)

Data are presented as mean ± standard deviation, median (range), or number (%), as appropriate.

Neonatal and obstetric outcomes are summarized in Table 2. Median gestational age was 40 weeks in both NC and FET–tNC groups, and 39 weeks in the FET–HRT group (*p* < 0.001; *η*^2^ = 0.041). Birth weight was slightly higher in FET–HRT infants (median 3,343.5 g) compared with NC (3,295.5 g) and FET–tNC (3,213 g), whereas birth length was marginally shorter in FET–HRT infants (50 cm vs. 51 cm). Caesarean rates were consistently high (70.5–77.1%) and did not differ significantly between groups (*p* = 0.469). The effect size for mode of delivery was very small (Cramer's *V* = 0.060), indicating negligible group influence.

**Table 2:** Comparative demographic data and neonatal outcomes across subgroups: naturally conceived (NC), FET–tNC (dydrogesterone), and FET–HRT (Est + dydrogesterone).

Variable	Naturally Conceived group (A) (*n* = 156)	FET–tNC group, Dydrogesterone (B) (*n* = 132)	FET–HRT group, Est + Dydrogesterone (C) (*n* = 144)	*p*-value	Effect size	Pairwise comparisons of groups
Age (year)	33 (30/36)	33 (30.5/35)	34 (30/40)	0.059^a^	*η*^2^ = 0.056	ns/ns/ns
BMI (kg/m^2^)	25.5 (23.5/29.9)	24.02 (21.7/26.5)	25.1 (23.4/27.9)	0.001^a^	*η*^2^ = 0.025	A vs. B: 0.001 A vs. C: 0.613 B vs. C: 0.053
Transfer P4 (ng/mL)	N/A	10.8 (7.0/21.4)	13.4 (9.5/28.6)	0.009^a^	*η*^2^ = 0.002	B vs. C: 0.006
Transfer E2 (pg/mL)	N/A	181 (155/333.5)	296 (229.5/334)	0.007^a^	*η*^2^ = 0.748	B vs. C: 0.006
TSH (mIU/L)	2.965 (2.1/4.0)	2.49 (1.8/3.3)	–	<0.001^a^	*η*^2^ = 0.014	A vs. B: N/A A vs. C: N/A B vs. C: 0.025
Birth length (cm)	51 (50/52)	51 (50/52)	50 (50/51)	0.001^a^	*η*^2^ = 0.087	A vs. C: 0.002
Birth weight (g)	3,295.5 (3,149.5/3,416)	3,213 (3,143.5/3,450)	3,343.5 (3,190/3,515)	0.003^a^	*η*^2^ = 0.050	B vs. C: 0.002
Birth week (week)	40 (39/41)	40 (39/41)	39 (38/40)	<0.001^a^	*η*^2^ = 0.373	B vs. C: 0.013
Anoscrotal distance (mm)	26.2 (25.5/27.2)	24.9 (24.1/25.6)	24.6 (23.8/25.6)	<0.001^a^	*η*^2^ = 0.399	A vs. B <0.001 A vs. C: <0.001 B vs. C: 0.354
Mode of delivery				0.469^b^	Cramer's V = 0.039	
Caesarean	116 (74.4)	93 (70.5)	111 (77.1)			NS/NS/NS
Vaginal birth	40 (25.6)	39 (29.5)	33 (22.9)			NS/NS/NS
Additional anomaly				0.030^b^	Cramer's V = 0.111	
Hypospadias	35 (22.4)	34 (25.8)	22 (15.3)			NS/NS/0.031
Undescended testis	24 (15.4)	10 (7.6)	13 (9.0)			0.041/NS/NS
Absent	97 (62.2)	88 (66.7)	109 (75.7)			NS/0.012/NS

Continuous variables are presented as median (IQR) with Kruskal–Wallis *p*-values and effect sizes (*η*^2^). Categorical variables are presented as *n* (%) with Pearson's *χ*^2^ *p*-values and effect sizes (Cramer's *V*).

Med., median; SD, standard deviation; Q1, 25th percentile; Q3, 75th percentile; ns, not significant.

a Kruskal–Wallis test (Monte Carlo); *post hoc* test: Dunn's test.

b Pearson Chi Square Test(Monte Carlo, Exact); *post hoc* Test: Benjamini–Hochberg correction.

*η*^2^ (eta-squared) values represent variance explained by group differences for continuous outcomes (small ≈ 0.01, moderate ≈ 0.06, large ≈ 0.14).

Cramer's *V* values quantify association strength for categorical comparisons (small ≈ 0.10, moderate ≈ 0.30).

**Figure 1:**
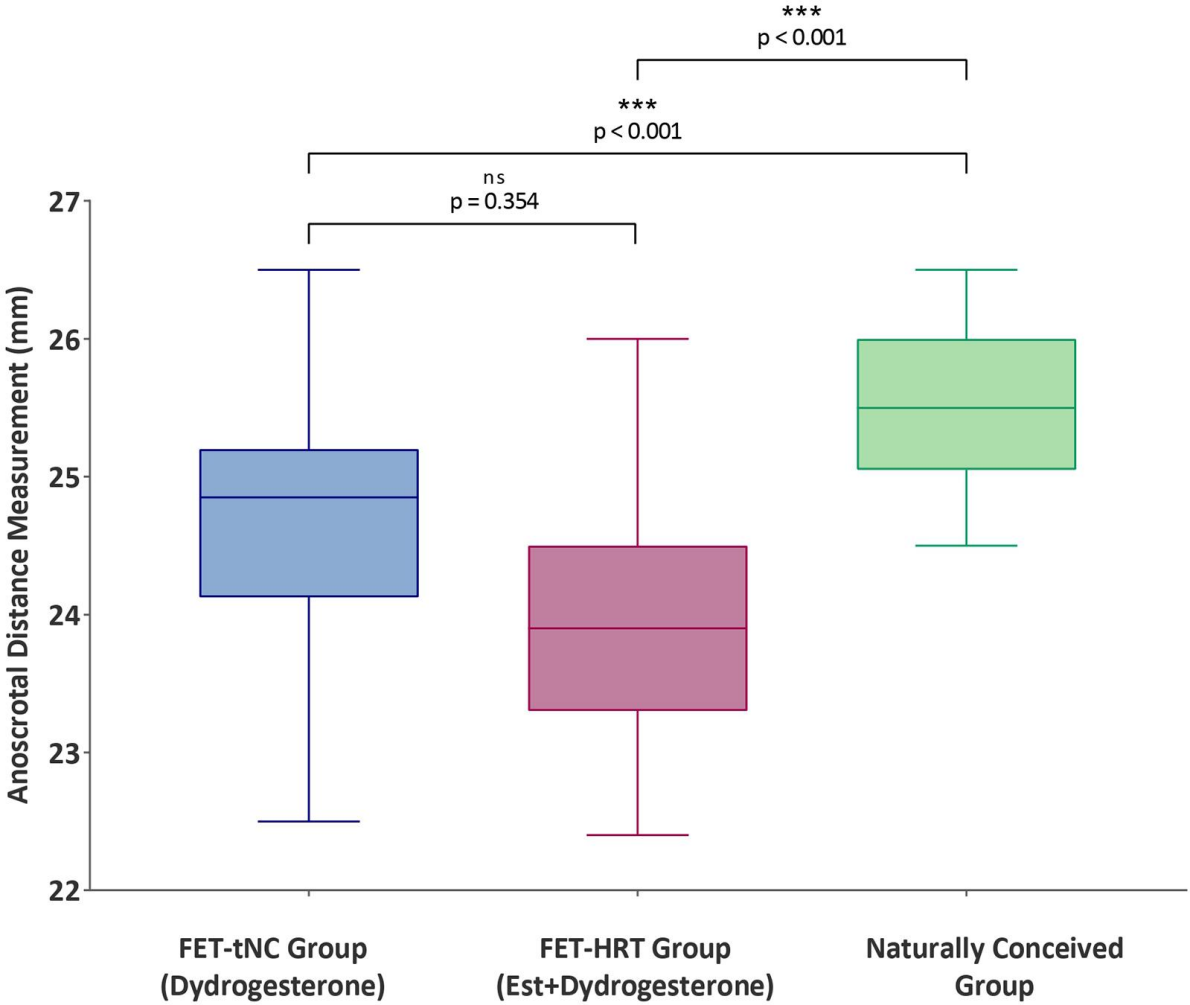
Comparison of neonatal anoscrotal distance (ASD) across conception groups.

The primary outcome, ASD, differed significantly across conception groups (*p* < 0.001, *η*^2^ = 0.067). NC infants had the longest ASD (median: 26.2 mm, IQR: 25.5–27.2), followed by FET–tNC (24.9 mm, IQR: 24.1–25.6) and FET–HRT (24.6 mm, IQR: 23.8–25.6). Pairwise testing confirmed that both FET groups had significantly shorter ASD than NC (*p* < 0.001 for each), while FET–tNC and FET–HRT did not differ from each other (*p* = 0.354) (Figure 1, Table 2). The absolute difference (≈1.3–1.6 mm) corresponds to a small-to-moderate effect size, indicating a consistent and measurable reduction in this androgen-sensitive anthropometric marker.

Categorical urogenital anomalies showed small effect sizes overall (Cramer's *V* = 0.111). Hypospadias prevalence differed across groups (*p* = 0.032, Cramer's *V* = 0.111), with a lower frequency in the FET–HRT group compared with FET–tNC (*p* = 0.031). Undescended testes were more common in NC infants compared with FET–tNC (*p* = 0.041), although no significant differences were observed between FET–tNC and FET–HRT. These associations, while statistically significant, correspond to small clinical effect sizes.

Stratification of ASD by birth-weight categories (<2,500 g, 2,500–3,000 g, 3,000–3,500 g, 3,500–4,000 g, ≥4,000 g) revealed a stable pattern: within every weight category, NC infants exhibited ASD values that were equal to or greater than those of both FET groups. Differences between FET–tNC and FET–HRT remained minimal. This indicates that birth weight did not modify the ASD differences observed between conception groups.

**Figure 2:**
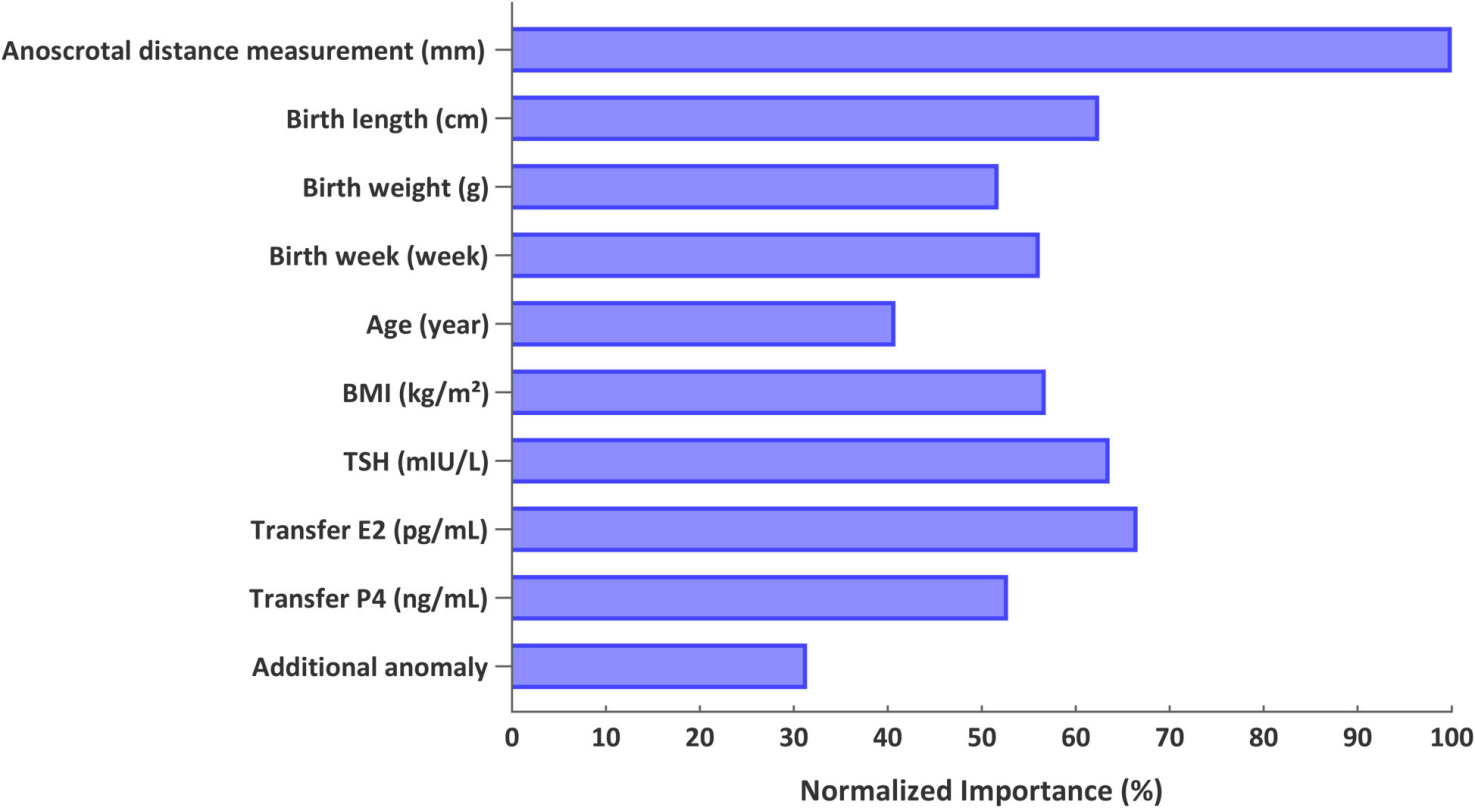
Analysis of subgroup contributions to prediction model performance based on demographic and neonatal outcomes.

Exploratory machine-learning analyses demonstrated that ASD was the strongest classifier of conception group, followed by transfer-day estradiol and TSH. Other neonatal metrics, such as birth length, BMI, and gestational week, contributed moderately, while additional anomalies accounted for the lowest predictive weight (Figure 2, Table 3).

**Table 3:** Predictive accuracy of demographic and neonatal parameters for subgroup classification, according to variable importance within the neural network model.

Variable Importance		Sample (Holdout)	Predicted			
Independent Variable	Normalized Importance		FET–tNC (Dydrogesterone)	FET–HRT (Est + Dydrogesterone)	Naturally Conceived (NC)	Percent Correct
Anoscrotal distance (mm)	100.00%	*Training (%70)*				
Transfer E2 (pg/mL)	66.60%	FET–tNC (Dydrogesterone)	71	4	16	78.00%
TSH (mIU/L)	63.60%	FET–HRT (Est + Dydrogesterone)	12	85	9	80.20%
Birth length (cm)	62.50%	Naturally Conceived (NC)	3	4	103	93.60%
BMI (kg/m^2^)	56.80%	Overall %	28.00%	30.30%	41.70%	84.40%
Birth week (week)	56.20%	*Testing (%30)*				
Transfer P4 (ng/mL)	52.80%	FET–tNC (Dydrogesterone)	26	8	7	63.40%
Birth weight (gr)	51.80%	FET–HRT (Est + Dydrogesterone)	6	22	10	57.90%
Age (year)	40.80%	Naturally Conceived (NC)	9	3	34	73.90%
Additional anomaly	31.40%	Overall %	32.80%	26.40%	40.80%	65.60%

Neural Network (Multilayer Perceptron), Hidden layer activation function: Hyperbolic tangent, Output layer activation function: Hyperbolic tangent, Dependent Variable: Groups.

## 4 Discussion

In this study, male infants conceived through FET, whether in a natural cycle (FET–tNC) or following hormone-replacement endometrial preparation (FET–HRT), exhibited significantly shorter neonatal ASD compared with those conceived naturally. The consistency of this finding across both ART protocols suggests that reduced ASD may be related to factors inherent to assisted reproduction or to underlying subfertility, rather than to the specific method of endometrial preparation.

ASD is a well-established anthropometric marker of prenatal androgen exposure, reflecting hormonal influences during the critical window of genital tubercle development in the first trimester (14). A reduction in ASD among ART-conceived male infants may therefore indicate alterations in the intrauterine hormonal milieu. Potential contributors include luteal phase support regimens, controlled ovarian stimulation in fresh cycles preceding cryopreservation, or intrinsic parental characteristics associated with infertility (14–17). Beyond these hormonal factors, emerging evidence suggests that ART procedures may influence epigenetic regulation during early embryogenesis, including altered DNA methylation and imprinting-related changes that could affect androgen-responsive developmental pathways (18–20). Additionally, differences in placental development and function observed in ART pregnancies such as altered trophoblast invasion, vascular remodeling, and steroidogenic activity, may modify fetal androgen exposure, providing a plausible mechanistic link between ART and reduced ASD (21, 22).

The absence of a significant difference between FET–tNC and FET–HRT in our cohort supports the interpretation that the ART process itself, rather than exogenous estrogen and progesterone used in HRT cycles, is the dominant factor. This further strengthens the concept that upstream processes—such as gamete handling, embryo culture, freezing–thawing procedures, and early epigenetic reprogramming—may exert a more substantial influence than the maternal hormonal preparation alone (23–25).

Secondary outcomes, including hypospadias and undescended testes UDT, demonstrated modest but inconsistent patterns across groups. The lower prevalence of hypospadias in the FET–HRT group compared with FET–tNC may be incidental, as no clear biological gradient was observed and similar findings have not been consistently reported elsewhere. Likewise, the higher prevalence of UDT in the NC group compared with FET–tNC contrasts with earlier evidence that ART pregnancies may increase the risk of cryptorchidism (2, 26). These inconsistent patterns highlight the multifactorial nature of congenital urogenital anomalies and the need for larger, adjusted analyses to separate treatment effects from background risk factors.

The literature examining the relationship between ART and ASD remains inconsistent. While some studies have reported shorter anogenital distances in ART-conceived male infants, others have found no significant differences after adjusting for maternal and perinatal characteristics (7, 27–29). Such discrepancies likely reflect heterogeneity in study populations, methodological variability in ASD measurement, and differences in underlying parental fertility status (14). Our findings align with reports demonstrating reduced ASD in ART-conceived male infants; however, these results should be interpreted with caution given the absence of detailed hormonal measurements during the critical early gestational window in our cohort.

Biologically, several mechanisms may plausibly link ART to subtle reductions in ASD, including altered placental steroidogenesis, epigenetic modifications arising from embryo culture or cryopreservation, and changes in early embryonic signaling pathways affecting androgen-sensitive development. Although the observed reduction in ASD (approximately 1.3–1.6 mm) appears modest in absolute magnitude, ASD is a highly sensitive biomarker of prenatal androgen action, and shorter measurements have been associated with increased risks of hypospadias, cryptorchidism, reduced spermatogenic potential, and lower circulating testosterone levels later in life (14). Whether the degree of shortening detected in our study predicts long-term reproductive or endocrine outcomes remains uncertain. Therefore, prospective longitudinal studies incorporating hormonal assessments, placental profiling, and follow-up into adolescence and adulthood are needed to determine the true clinical significance of these early anthropometric differences in ART-conceived males.

The strengths of this study include its relatively large sample size, standardized measurement of ASD within 48 h of birth, and direct comparison of two widely used FET protocols with a natural-conception reference group. Limitations include the retrospective, single-centre design and the absence of formal inter-observer reproducibility testing for ASD measurements. Several clinically relevant confounders such as maternal smoking status, parity, environmental exposures, paternal reproductive characteristics, parental age, specific causes of infertility, and details of embryo culture and laboratory conditions were not consistently recorded in the medical files and therefore could not be incorporated into the analyses. Because these variables were unavailable, a fully adjusted multivariable regression model could not be performed, and the possibility of residual confounding cannot be excluded. Additionally, subgroup sample sizes were modest, raising the potential for Type II error and limiting the precision of non-significant comparisons.

Future work should include prospective cohort studies with detailed hormonal profiling across early gestation, mechanistic evaluations of placental and epigenetic pathways, and long-term follow-up of ART-conceived males to determine whether neonatal ASD differences persist and carry implications for pubertal development or adult reproductive health.

## 5 Conclusion

In summary, male infants conceived through FET—regardless of protocol—consistently show shorter neonatal ASD compared with naturally conceived infants. As ASD is a marker of androgen-sensitive genital development, these findings highlight the need for closer surveillance of male genital outcomes in ART-conceived offspring. Given the consistency of ASD reduction across FET protocols and growing evidence linking early anogenital measurements to later reproductive health, clinicians may consider incorporating routine, standardized assessment of ASD and genital development into postnatal evaluations of ART-conceived male infants. Such early screening may facilitate timely recognition of atypical androgen exposure, guide decisions regarding follow-up, and contribute to improved long-term reproductive health surveillance.

## Data Availability

The raw data supporting the conclusions of this article will be made available by the authors, without undue reservation.
